# Exosomes enriched in stemness/metastatic-related mRNAS promote oncogenic potential in breast cancer

**DOI:** 10.18632/oncotarget.5818

**Published:** 2015-10-26

**Authors:** Marta Rodríguez, Javier Silva, Alberto Herrera, Mercedes Herrera, Cristina Peña, Paloma Martín, Beatriz Gil-Calderón, María Jesús Larriba, Mª José Coronado, Beatriz Soldevilla, Víctor S. Turrión, Mariano Provencio, Antonio Sánchez, Félix Bonilla, Vanesa García-Barberán

**Affiliations:** ^1^ “Mecanismos Moleculares Tumorales” Research Group, Department of Medical Oncology, IDIPHIM, Instituto de Investigación Sanitaria Puerta de Hierro, Madrid, E-28222, Spain; ^2^ “Señalización Celular en Cáncer” Research Group, Department of Medical Oncology, IDIPHIM, Instituto de Investigación Sanitaria Puerta de Hierro, Madrid, E-28222, Spain; ^3^ Laboratory of Molecular Pathology, Department of Pathology, IDIPHIM, Instituto de Investigación Sanitaria Puerta de Hierro, Madrid, E-28222, Spain; ^4^ Instituto de Investigaciones Biomédicas “Alberto Sols”, Department of Cancer Biology, CSIC-UAM, Madrid, E-28029, Spain; ^5^ Confocal Microscopy Core Facility, IDIPHIM, Instituto de Investigación Sanitaria Puerta de Hierro, Madrid, E-28222, Spain; ^6^ Department of Molecular Biology, Centro de Biología Molecular Severo Ochoa, Consejo Superior de Investigaciones Científicas-Universidad Autónoma de Madrid, Centro de Investigación Biomédica en Red de Enfermedades Raras CIBERER-ISCIII, Madrid, E-28049, Spain; ^7^ “Diagnóstico y pronóstico molecular en cáncer” Research Group, Department of Medical Oncology, IDIPHIM, Instituto de Investigación Sanitaria Puerta de Hierro, Madrid, E-28222, Spain; ^8^ Department of Digestive and General Surgery, Hospital Universitario Puerta de Hierro Majadahonda, Madrid, E-28222, Spain; ^9^ Department of Medical Oncology, Hospital Universitario Puerta de Hierro Majadahonda, Madrid, E-28222, Spain; ^10^ Centro de Estudios Biosanitarios, Madrid, E-28029, Spain; ^11^ Molecular Oncology Laboratory, Department of Medical Oncology, IDISSC, Instituto de Investigación Sanitaria San Carlos, Madrid, E28040, Spain

**Keywords:** exosomes, liquid biopsy, breast cancer, mRNA, stemness and metastasis

## Abstract

Cancer cells efficiently transfer exosome contents (essentially mRNAs and microRNAs) to other cell types, modifying immune responses, cell growth, angiogenesis and metastasis. Here we analyzed the exosomes release by breast tumor cells with different capacities of stemness/metastasis based on CXCR4 expression, and evaluated their capacity to generate oncogenic features in recipient cells. Breast cancer cells overexpressing CXCR4 showed an increase in stemness-related markers, and in proliferation, migration and invasion capacities. Furthermore, recipient cells treated with exosomes from CXCR4-cells showed increased in the same abilities. Moreover, inoculation of CXCR4-cell-derived exosomes in immunocompromised mice stimulated primary tumor growth and metastatic potential. Comparison of nucleic acids contained into exosomes isolated from patients revealed a “stemness and metastatic” signature in exosomes of patients with worse prognosis. Finally, our data supported the view that cancer cells with stem-like properties show concomitant metastatic behavior, and their exosomes stimulate tumor progression and metastasis. Exosomes-derived nucleic acids from plasma of breast cancer patients are suitable markers in the prognosis of such patients.

## INTRODUCTION

Exosomes are a type of nanovesicles that differ from other extracellular vesicles in their endocytic origin, their size (40–100 nm) and their specific molecular cargo. Exosomes released from cells, including tumor cells, provide a significant mechanism of intercellular communication. Previous studies in cancer cells have shown that these released nanovesicles transfer their content to other cell types, modifying immune responses, cell growth, angiogenesis and metastasis [1, 2].

Breast cancer is the most commonly diagnosed cancer in women [3], and metastasis is responsible for morbidity and the majority of cancer-related death. Metastasis is a multi-step evolutionary process in which cancer cells acquire alterations allowing them to transcend their programmed behavior to disseminate from the primary tumor, intravasate into the blood circulation and eventually extravasate into foreign tissues. Ultimately, a few of these cells (0.01–0.02%) will adapt to the new distal microenvironment and form macrometastases [4]. Moreover, only a subpopulation of cancer cells with stem-like properties is competent to initiate tumor growth and disseminate to distant organs [5, 6]. These cancer stem cells (CSC) are able to self-renew and generate the heterogeneous lineages of cancer cells that comprise the tumor. Furthermore, these cells have phenotypic plasticity and resist drug-induced DNA damage. Collectively, the capacity of these cells to evade destruction and survive at distal sites makes CSC more likely to support the establishment of primary tumors and to succeed in the later steps of metastasis. In addition, this property may explain why micrometastases can remain dormant after removal of the primary tumor and recur many years later [7, 8]. Thus, these CSCs are responsible for metastatic growth in breast cancer which contributes to majority of the breast cancer related morbidity and mortality.

Recently, many studies have shown that the presence of the chemokine receptor CXCR4 is involved in many stages of tumorigenesis, as invasion and metastasis in several cancers, including breast cancer [9]. The paracrine and endocrine effects through CXCL12/CXCR4 are critical for tumor growth, invasion, angiogenesis and metastasis [10]. This axis is also involved in cancer stem cell characteristics [11, 12], and is an important marker for metastatic potential of CSC [13]. Furthermore, increased CXCR4 is correlated with high risk for recurrence and poor overall survival in multiple cancer patients including breast, lung, kidney, colon, ovarian, and brain cancers, as well as lymphoma and leukemia.

There is therefore a need to better understand mechanisms associated with metastatic process in breast cancer. We used a model based on CXCR4-transfected culture to analyze the role of exosomes released from breast tumor cells with different stemness/metastasis abilities in the capacities for tumor growth, generation of stem cell features in neighboring cells, and metastatic potential. Finally, exosomes from plasma of breast cancer patients were characterized and assessed their capacity as prognostic marker in liquid biopsy.

## RESULTS

For this study, we used the expression of aldehyde dehydrogenase (ALDH) to identify cancer cells with stem cell features in three human breast cancer cell lines. Similarly, as described elsewhere [14], HCC38 cell line showed 100% of ALDH-positive cells, whereas MDA-MB-231 and T47D cell lines showed 1% and 0% of ALDH-positive cells respectively (Supplementary Figure S1A). Therefore, T47D cells showed absence of stem cell features, and previously, it has been broadly identified as non-invasive and non-metastasic, forming tumors only in the presence of oestrogen [15]. For these reasons, this cell line was selected to stably transfect CXCR4 gene (Supplementary Figure S1B) and to study the paracrine effect of its exosomes when CXCR4 is overexpressed. HCC38 and MDA-MB-231 cell lines were used as controls of stemness and metastatic capacities, respectively.

Before of analyse the potential effects of exosomes released by CXCR4-cells, we tested the effects of CXCR4 overexpression in the transfected breast cancer cell line. As expected, CXCR4 expression increases proliferation, migration and invasion capabilities *in vitro* (Supplementary Figure S2A–S2C). Moreover, contribution of CXCR4 to tumorigenic and metastatic capacities was observed in two *in vivo* models: using a tumorogenic/metastatic cell line (MDA-MB-231) and a non-tumorogenic cell line (T47D) (Supplementary Figure S2D–S2G). Moreover, CXCR4-cells showed increased expression of stemness-related markers (Supplementary Figure S3). Together, these results using *in vitro* and *in vivo* models indicate that expression of CXCR4 stimulates tumorogenic and metastatic capacities in breast cancer cells, confirming previously described data.

### Exosomes released by CXCR4-cells increase, by a paracrine manner, stemness-related markers expression, proliferation, migration and invasion *in vitro* in breast cancer cells

Correct exosomes isolation were confirmed by transmission electron microscopy (Supplementary Figure S4A) and nanoparticle tracking analysis (Supplementary Figure S4B), which revealed vesicles within the expected size range (50–100 nm). Moreover, immunoblotting confirmed the presence of exosomal proteins CD63 and CD81 and the absence of negative control Calnexin (Supplementary Figure S4C). To analyze the uptake of exosomes by recipient cells, T47D-CXCR4 exosomes were labelled with PKH67 dye and added them to cultures of T47D cells. Confocal microscopy confirmed the internalization of T47D-CXCR4 exosomes in T47D cells (Supplementary Figure S4D).

**Figure 1:**
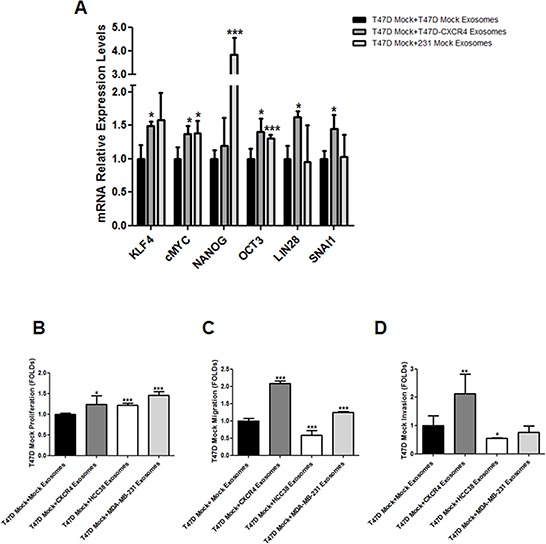
A. mRNA expression of stemness-related markers in recipient cells after addition of exosomes released by CXCR4-transfected cells **B.** Proliferation assay, **C.** migration assay and **D.** invasion assay in recipient cells after addition of exosomes released by CXCR4-transfected cells, HCC38 and MDA-MB-231 cells. *p ≤ 0.05; **p ≤ 0.01; and ***p ≤ 0.005.

Expression of genes involved in stemness was quantified in T47D recipient cells after addition of T47D-CXCR4 exosomes. As the epithelial-mesenchymal transition (EMT) has been implicated in the generation of stem cell properties [16], expression of *SNAI1* and *CDH1* was also quantified. We found increased expression of stemness- and EMT-related mRNAs in T47D cells after addition of T47D- and MDA-MB-231-CXCR4 exosomes (Figure 1A).

After addition of exosomes from T47D-CXCR4 cells, T47D cells exhibited higher proliferation capacity than T47D cells incubated with equal amount of exosomes from T47D mock cells. Similarly, T47D cells incubated with MDA-MB-231- and HCC38-exosomes showed increased proliferation with respect to control cells (Figure 1B). T47D cells incubated with exosomes from T47D-CXCR4 and MDA-MB-231 showed increased migration potential with respect to T47D cells incubated with T47D-mock exosomes (Figure 1C). In contrast, T47D cells incubated with exosomes from HCC38 cells showed lower migration capacity. Similarly, T47D cells incubated with exosomes from T47D-CXCR4 showed increased invasion with respect to cells incubated with T47D-mock exosomes. T47D cells incubated with exosomes from HCC38 cells showed lower invasion capacity (Figure 1D).

These results indicate that exosomes derived from CXCR4-tumor cells modify stemness markers, proliferation, migration and invasion features of neighbouring cells.

### Exosomes released by CXCR4-cells increase the oncogenic potential of tumor cells in mice

Next, roles of CXCR4-cells-derived exosomes in primary tumor growth and metastatic capacity were examined in two animal models: a tumorogenic/metastatic model using MDA-MB-231 cell line, and a non-tumorogenic model using T47D cell line.

**Figure 2:**
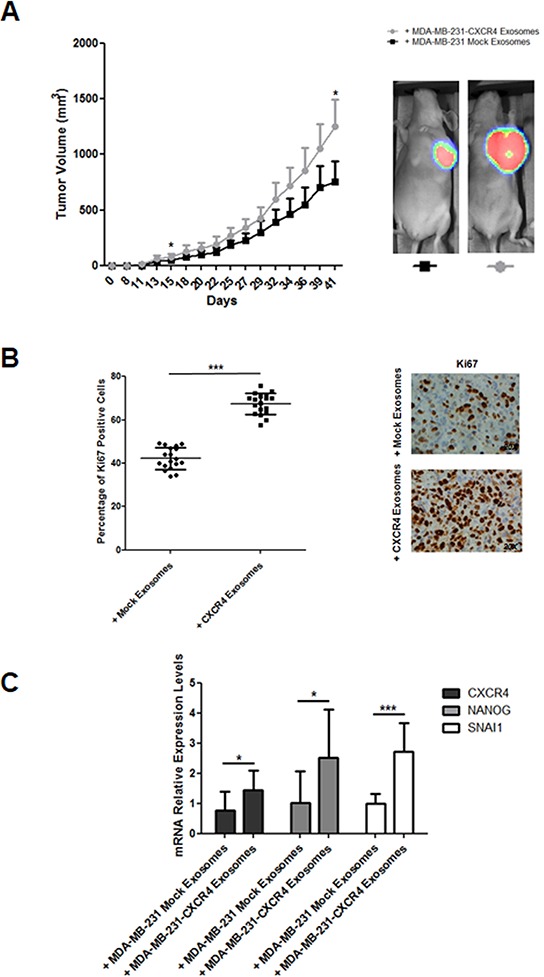
A. Evolution of tumor growth in immunodeficient mice intravenously injected with MDA-MB-231-CXCR4-derived exosomes *In vivo* ventral coelenterazine-based F^Luc^-BLI images of a representative mouse (*p ≤ 0.05). **B.** Ki67 immunohistochemistry of primary tumors developed in immunodeficient mice intravenously injected with MDA-MB-231-CXCR4-derived exosomes (***p ≤ 0.005). **C.** *CXCR4*, *NANOG* and *SNAI1* mRNA levels in primary tumors developed in immunodeficient mice intravenously injected with MDA-MB-231-CXCR4-derived exosomes as compared with tumors in mice treated with MDA-MB-231-mock-derived exosomes (*p ≤ 0.05; and ***p ≤ 0.005).

**Figure 3:**
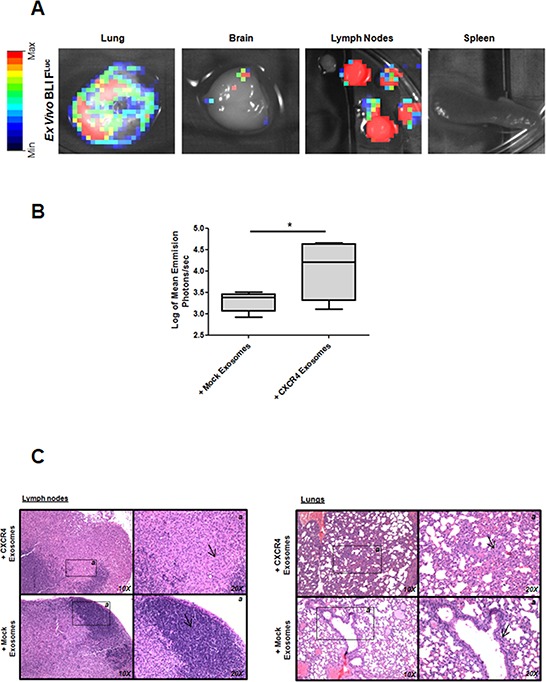
Metastasis after fat pad injection of MDA-MB-231^FLuc^ cells in mice treated with exosomes **A.** *Ex vivo* D-luciferin-based F^Luc^-BLI images of excised lung, brain, lymph nodes and spleen of a representative mouse. **B.** Quantification of total photon flux in lymph nodes (*p ≤ 0.05). **C.** Hematoxylin and eosin-stained sections taken from different part of lymph nodes and lungs (10X and 20X images are shown).

In the first model, ten mice were treated with MDA-MB-231-CXCR4- or mock-exosomes intravenously injected, starting one day after orthotopic injection of MDA-MB-231^FLuc^ cells. Cells with firefly luciferase (F^Luc^) reporter gene were used to improve the evaluation of metastasis. In mice injected with MDA-MB-231-CXCR4 exosomes, primary tumors showed enhanced tumor growth (Figure 2A), high percentage of Ki67 positive cells (Figure 2B) and increased levels of stemness/EMT-related mRNAs (Figure 2C). In addition, more metastasis was detected by *ex vivo* Bioluminescent Imaging (BLI) in mice treated with CXCR4-exosomes (Figure 3A): lymph nodes (ten mice), lung (three mice) and brain (one mouse) (Figure 3B). With mock-exosomes treatment, metastasis was only detected in lymph nodes of nine mice. Metastatic lesions were confirmed by immunohistochemistry using haematoxylin and eosin stain (Figure 3C).

Other mouse model was performed based on non-tumorogenic/metastatic cell line (T47D). Interestingly, mice treated with T47D-CXCR4 exosomes showed lymph node micrometastasis (Supplementary Figure S5).

These data indicate that exosome cargo from CXCR4-cells enhance tumor growth and metastatic potential in breast cancer models.

### Exosomes released by T47D-CXCR4 are enriched in mRNAs related with stemness and metastasis

Trying to identify the possible mediators involved in the cross-talk between different cells through exosomes, the genetic information contained in exosomes released by T47D-CXCR4 cells was analyzed by PCR arrays. Thus, PCR arrays with most representative mRNAs involved in metastasis and stem cell differentiation and development were performed in T47D-CXCR4 exosomes *versus* T47D-mock exosomes and HCC38 exosomes. T47D-CXCR4 exosomes were highly enriched in genes related with stem cell differentiation and development. Similarly, exosomes isolated from HCC38 cells (100% ALDH-positive) were highly enriched in these types of mRNA (Supplementary Figure S6). Thus, CXCR4 expression is involved in exosomes-gene expression profile changes related to biological process enrichment found in tumor cells with high stemness potential. The top ten enriched mRNAs carried in exosomes are shown in Supplementary Table S1.

### Stemness and metastasis-related mRNAs contained in exosomes from plasma patients with breast cancer are associated with poor prognosis

In the same way, mRNAs related with stemness and metastasis were compared in pools of exosomes from plasma of patients by PCR array analysis. Patients were divided in two pools in base of their outcome: “Good outcome” and “Poor outcome” pool. Good outcome pool included patients without first relapse and death, and large follow-up (median follow-up of 119 months). Poor outcome pool included patients with first relapse and death, and short survival (median follow-up of 17 months). Levels of 8 metastasis-related mRNAs and 27 stemness-related mRNAs were higher in exosomes from patients with poor prognosis than in exosomes from patients with good prognosis. Supplementary Figure S6 shows the number of mRNAs with high levels detected in T47D-CXCR4 and HCC38 cells, and in the pool of patients with poor prognosis. mRNAs with highest levels detected in exosomes of poor prognosis pool (*NANOG*, *NEUROD1*, *HTR7*, *KISS1R*, *HOXC6*) were also increased in exosomes released by T47D-CXCR4 and HCC38 (Supplementary Table S1). These mRNAs were analyzed by real-time PCR in a large series of plasma from 173 breast cancer patients.

**Figure 4:**
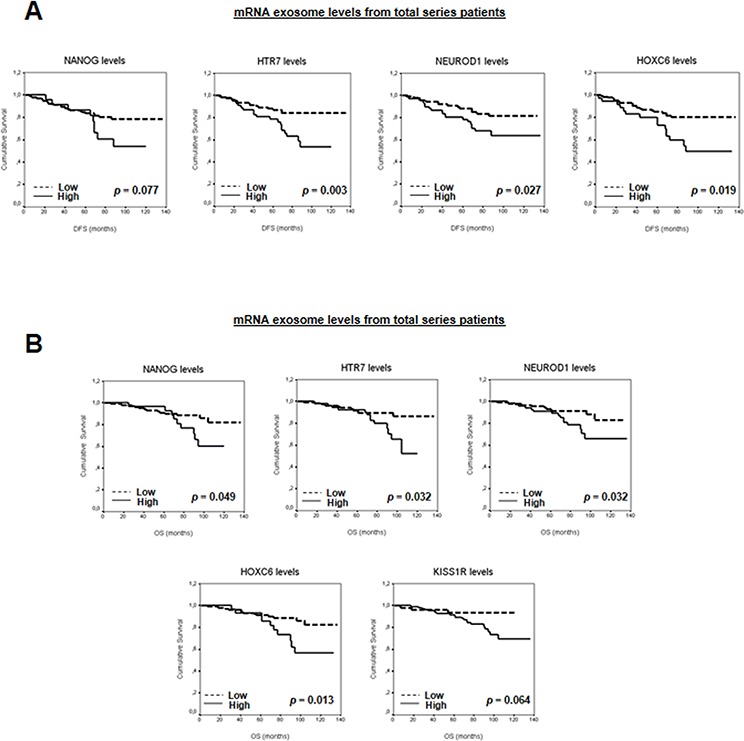
A. Kaplan-Meier DFS curves in relation to levels of validated mRNA in exosomes from total series patients **B.** Kaplan-Meier OS curves in relation to levels of validated mRNA in exosomes from total series patients.

The patient series was followed for a mean of 73 months (range: 1–136). Individual survival analysis of each selected mRNA showed associations between high mRNA levels and patients DFS or OS (Figure 4 and Supplementary Table S2A). When levels of mRNA in exosomes from plasma were stratified for hormone receptors and HER2, associations were found between poor outcome in patients and high mRNA levels in exosomes in hormone-positive receptors and HER2-negative subgroups (Supplementary Figure S7 and Supplementary Table S2B–S2C). Moreover, high *NANOG* mRNA levels were associated with short DFS in the HER2-positive subgroup.

**Figure 5:**
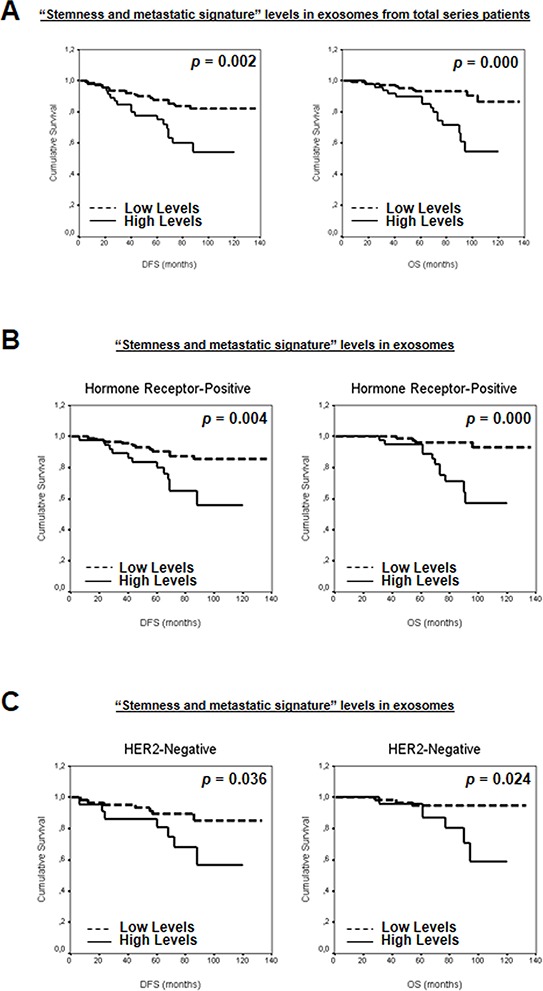
**Kaplan-Meier DFS and OS curves in relation to “stemness and metastatic signature” levels in exosomes from A.** total series patients, and patients classified on the basis of **B.** hormone receptors and **C.** HER2 status.

To study the possible additive effect of the selected mRNAs as a “stemness and metastatic signature”, the patients were grouped according to the number of mRNA with high levels in plasma exosomes. Thus, patients were first classified on the basis of high levels of zero, one, two, three, four or five mRNAs. A clear association was observed between increased exosome-derived nucleic acids and breast cancer patients DFS or OS (*P* = 0.021 and *P* = 0.016, respectively). Next, to dichotomize the analysis, and based on similar mathematical behavior, patients with high levels of zero, one or two mRNAs were grouped as “low-levels” and those with more than three mRNAs were grouped as “high-levels”. Interestingly, correlation between DFS or OS previously observed was stronger (Figure 5A and Supplementary Table S3). Adjusted analysis showed that this “stemness and metastatic signature” in exosomes had an independent relationship with OS and a trend with DFS (Table 1). In hormone receptor-positive and HER2-negative breast cancers, high levels of this signature were significantly associated with shorter DFS and OS (Figure 5B–5C and Supplementary Table S3). Moreover, relationships were found between clinicopathological parameters and signature high levels in exosomes (Supplementary Table S4).

**Table 1:** Multivariate cox analysis of the association between “stemness and metastatic signature” and DFS and OS of breast cancer patients

	Variable	Category	Adjusted Analysis		*P* Value
			Hazard Ratio	95% CI	
**DFS**	**PR**	Positive *vs* Negative	0.28	0.11–0.74	0.010
	**HER2**	Positive *vs* Negative	3.53	1.2–10.2	0.020
	**RT**	progression *vs* complete	23.13	5.05–106.08	<0.001
	**Signature level**	High *vs* Low	2.68	0.95–7.53	0.062
**OS**	**VLI**	Yes *vs* No	5.52	2.03–15.04	0.001
	**HR**	Positive *vs* Negative	0.16	0.06–0.42	<0.001
	**RT**	progression *vs* complete	18.15	2.04–161.65	0.009
	**Signature level**	High *vs* Low	5.77	2.23–14.95	<0.001

VLI, vascular and lymphatic invasion; PR, progesterone receptor; HR, hormone receptors; RT: response to treatment.

Together, these results support the involvement of “stemness and metastatic” signature in exosome-derived nucleic acids in breast cancer patient survival.

## DISCUSSION

In summary, this study demonstrates how exosomes derived from CXCR4-breast tumor cells modify stemness markers and enhance proliferation, migration and invasion abilities of neighboring cells. The *in vivo* significance of these findings is documented by the observation that inoculation of CXCR4-cells-derived exosomes in immunocompromised mice enhanced primary tumor growth and metastatic potential. Furthermore, clinical relevance is indicated by the observation that comparative analysis of mRNAs contained in exosomes isolated from patients revealed a gene signature of nucleic acids highly enriched in exosomes of patients with worse prognosis.

Exosomes influence most tumor-related pathways, such as proliferation, angiogenesis, EMT, promotion of immune escape, cancer stemness and metastasis involving many cell types within the tumor microenvironment [2, 17]. Regarding the stemness and the dissemination potential, cancer stem cell and metastatic cells share comparable behaviour and properties such as the capacity for self-renewal, the requirement for a specific microenvironment to grow and the SDF1/CXCR4 axis [18]. In line with the proposal that CSC shape the disseminating subpopulation of tumor cells, the positive correlation between EMT and CSC properties supports the theory of “migrating cancer stem cells” as the basis of metastasis colonization [19]. Numerous studies have reported that CSC also express EMT-related markers, and more crucially, that induction of EMT promotes the generation of CSC in transformed epithelial cells [18, 20]. Our study focused on these parallel characteristics in several culture models, based on the transfection of CXCR4, a widely accepted chemokine receptor implicated in metastasis of breast cancer cells that also is overexpressed in breast cancer stem cells (CSC) [21, 22]. The main goal of the study is the contribution of exosomes released from CXCR4-breast tumor cells to tumor growth, dissemination capacity and generation of stem cell features after uptake by recipient cells.

In concordance with roles of CXCR4 described above, CXCR4-cells showed well known EMT-related features, elevated expression of several stem cell-related genes and surface markers, and higher proliferation rates, migration activity and cell invasiveness than mock cells. These results are in line with others studies performed in breast cancer cell lines transfected with CXCR4 [23]. Interestingly, our data indicate that this observation was supported by analysing the contribution of CXCR4-cell-derived exosomes to tumorigenic capacity in acceptor cells. Collectively, the *in vitro* set of developed experiments showed that CXCR4-cells-derived exosomes entailed an increase in stemness- and EMT-related markers and induced proliferation, migration and invasion capacities in non-pluripotent/non-invasive recipient cells.

CXCR4 overexpression promoted metastatic potential and oestrogen independence tumor growth *in vivo*, similarly to previous studies performed in MCF7 cells. Thus, tumor stem-like cells show metastatic behaviour [5, 6]. Accordingly, our *in vivo* experiments in immunocompromised mice inoculated with CXCR4-cell-derived exosomes established that exosomes increased primary tumor growth and metastatic potential. Together, these findings indicate that exosomes released by cancer cells with parallel stem-like and metastatic properties transfer these oncogenic features to recipient cells.

Our study identified a specific T47D-CXCR4-derived exosome signature highly enriched in nucleic acids related to both stem cell differentiation and metastasis. Interestingly, this signature was highly homologous to the content of exosomes derived from HCC38, a cell line strongly positive for markers related with breast cancer stem cell subpopulations.

Moreover, by comparing the nucleic acids into exosomes from plasma of breast cancer patients divided by poor and good prognostic, several stemness and metastatic-related mRNAs in exosomes were identified, which were also increased in CXCR4-exosomes. Thus, we observed that levels of these mRNAs in exosomes are related to poor DFS and OS. When “stemness and metastatic” signature is taken into consideration, this relationship is more robust. Moreover, multivariate analysis clearly demonstrates that this variable is an independent prognostic marker for breast cancer patients. The selected exosome-derived nucleic acids have been implicated in growth and proliferation, induced pluripotent and embryonic stem cells, somatic stem cell maintenance, embryonic development, symmetry and segmentation, and in organ morphogenesis (Kyoto Encyclopedia of Genes and Genomes (KEGG) v58.1). The clinical relevance of these findings is established by the definition of a “stemness and metastatic signature” in exosomes, which showed a remarkable prognostic value for the clinical outcome of breast cancer patients. This finding could help predict the clinical outcome and inform decisions about treatment, mainly in hormone receptor-positive or HER2-negative breast cancer subgroups.

Here we propose that the communication through exosomes between cancer and surrounding cells stimulate tumor progression and metastasis, particularly when exosomes are released by breast cancer cells with stemness and metastatic properties. Our study reveals that CXCR4-exosomes promote breast cancer cells proliferation, motility and metastasis, generating an enhanced tumorigenesis phenotype. Furthermore, our findings contribute to the identification of a breast cancer prognostic signature, which could be easily translated into clinical practice since these markers are obtained by a non-invasive method. This signature could also be used during patients' follow-up to identify patients with worse prognosis. This data exemplify the ongoing efforts to understand the involvement of stemness and metastatic-related nucleic acids contents in exosomes in cancer progression. Search for new specific therapies against these exosomes would offer a synergistic effect with current therapies in those patients usually with worse outcome. In this way, CXCR4-related stemness properties displayed by tumor cells with metastatic capacity could be potentially exploited for therapeutic targeting of CSC using treatment anti-CXCR4 (e.g. TN14003 or AMD3100), also avoiding the effects of their exosomes.

## MATERIALS AND METHODS

### Cell culture

Cell lines were obtained from American Type Culture Collection. For culture conditions details, see Supplementary Material.

T47D and MDA-MB-231 cells were stably transfected with Precision LentiORF expression vectors encoding CXCR4 or RFP control particles (Thermo Scientific), using Lipofectamine 2000 (Invitrogen). One week post-transfection, CXCR4 and RFP-mock expressing cells were selected by sorter and expanded in RPMI supplemented with 20% FBS. MDA-MB-231^FLuc^ cells [24] were kindly provided by Dr. L. Vallina (University Hospital Puerta de Hierro Research Institute, Madrid, Spain).

### Exosome isolation, identification and quantification

Cell lines were cultured in media supplemented with exosome-depleted FBS. FBS was depleted of bovine exosomes by passage through a 0.22-μm PVDF filter (Millipore) and ultracentrifugation at 120,000 *g* for 90 minutes. Exosomes were isolated from cell supernatants by a series of centrifugation and filtration steps (see Supplementary Material). The exosome pellet was resuspended in 200 μL of phosphate-buffered saline (PBS) or medium with 1% exosome-depleted FBS. Circulating exosomes were isolated from human plasma with ExoQuick™ plus Thromboplastin D Kit (System Bioscience), in line with the manufacturer's instructions.

For identification by transmission electron microscopy (model JEOL Jem1010, 100 kV) exosomes were fixed in 2% PFA (w/v) in 200 mM phosphate buffer (pH 7.4). Fixed exosomes were dropped onto a formvar-carbon-coated grid and left to dry at room temperature for 20 minutes. After washing in PBS, the exosomes were fixed in 1% glutaraldehyde for 5 minutes, washed in water and stained with saturated aqueous uranyl oxalate for 5 minutes. Samples were then embedded in 0.4% (w/v) uranyl acetate and 1.8% (w/v) methylcellulose and incubated on ice for 10 minutes. The excess liquid was then removed. The grid was dried at room temperature for 10 minutes and viewed at 80,000 and 120,000 magnification.

LM10 nanoparticle characterization system (NanoSight) was used for real-time characterization and quantification of the vesicles in supernatant fractions and in samples from patients.

### Exosome labeling

For the exosome-tracking experiments, purified exosomes from cell lines were fluorescently labeled using PKH67 membrane dye (Sigma-Aldrich) following the manufacturer's instructions. Images were collected with a TCS SP5 confocal microscope (Leica Microsystems). For details, see Supplementary Information.

### Protein extraction and western blot analysis

Exosomes were lysed with T-PER Tissue Protein Extraction buffer (Thermo Scientific) containing a protease and phosphatase inhibitor cocktail (Thermo Scientific). Western Blots for CD63 (ab59479, Abcam), CD81 (349502, BioLegend Inc.) and Calnexin (endoplasmic reticulum protein) as loading control (sc-11397, Santa Cruz Biotechnology) were performed. For details, see Supplementary Information.

### RNA extraction, retrotranscription and mRNA quantitative analysis

Total RNA was extracted from cells and exosomes by the *mir*Vana™ miRNA Isolation Kit (Ambion Inc.). Retrotranscription and amplification were performed using Transcription first-strand cDNA Synthesis Kit and LightCycler 480 SYBR Green I Master Kit in a Light-Cycler apparatus (Roche Diagnostics). For PCR Arrays analysis we used RT^2^ PreAMP CDNA Synthesis Kit and RT^2^ PreAMP Pathway Primer Mix for Stem Cell Transcription Factors and for Human Tumor Metastasis, in the appropriate PCR Arrays following the manufacturer's instructions (Qiagen). In the validation set, retrotranscription was achieved as described above. Next, we performed a specific pre-amplification using Real-Time Ready cDNA PreAMP Master using PreAMP Primer Pools, followed by Real-Time Ready Custom Panels in a Light-Cycler apparatus (Roche Diagnostics) in line with the manufacturer's instructions.

cDNAs were quantified by NanoDrop ND-1000 (Thermo Scientific); and concentrations were equalized to 100 μg/μL to standardize per cDNA amount.

The sequences of the primer sets and the reaction conditions are shown in Supplementary Table S5.

### Proliferation assay

Proliferation assay was performed by seeding cell lines (20,000 cells/well) in 96-well E-plates in RPMI containing 1% exosome-depleted FBS for 24 and 48 hours. Similarly, T47D mock cells were incubated for 24 and 48 hours with CXCR4 or mock breast tumor-derived exosomes, and viable cells were detected using the MTT Cell Proliferation Assay Kit (Cayman Chemical Company). Absorbance was measured on a microplate reader at 570 nm (Multiskan Ex; Thermo Scientific).

### Migration and invasion assays

Tumor cells (100,000 cells/well) were cultured using a 24-well E-plates Transwell with 8.0-μm pore polycarbonate membrane inserts (Costar, Corning Inc.) in RPMI containing 1% exosome-depleted FBS. For invasion assays, Matrigel was applied to the upper surface of the membranes of Transwells (BD Biosciences). Similarly, T47D mock cells were incubated with CXCR4 or mock breast tumor-derived exosomes. Migration and invasion rates were analyzed by CellTiter-Glo^®^ Luminiscent Cell Viability Assay (Promega Corp.) and fluorescence reader (Tecan Infinite 200 Pro).

### Flow cytometry

Activity of ALDH was analyzed in cell lines using ALDEFLUOR assay (Stemcell Technologies) following the manufacturer's instructions. FACS analysis of surface antigens CD44 and CD49f was performed to identified subpopulation of breast cancer stem cells [25] (details in Supplementary Material). Data were acquired in a MACSQuant Analyzer using the MACSQuantify™ Software version 2.5 (Miltenyi Biotec S.L.). Results reported as percentage of positive cells for ALDH and as mean fluorescence intensity (MFI) for CD44 and CD49f.

### Animals and tumor xenografts

All experimental procedures were performed in accordance with the Spanish Government guidelines for the care and use of laboratory animals and were approved by the Hospital Universitario Puerta de Hierro Animal Care and Use Committee (CEBA).

Five-week-old female athymic nude mice (Hsd: Athymic Nude-Foxn1nu mice, Harlan Laboratories, Barcelona, Spain) were used for xenograft experiments. For the analysis of tumorigenesis, we injected 1 × 10^6^ T47D-CXCR4 or T47D mock cells and 1 × 10^6^ MDA-MB-231-CXCR4 or MDA-MB-231 mock cells suspended in a 20% Matrigel matrix (Matrigel™ Basement Membrane Matrix, BD Biosciences) into the second left mammary fat pad of each mouse. Tumors were measured three times per week using precision calipers. Tumor volume was calculated as width^2^ × length × 0.52. To analyze the role of exosomes in tumor growth and metastasis, 1 × 10^6^ MDA-MB-231^FLuc^ cells were injected into the mammary fat pad. For exosome treatment, 20 μg of CXCR4 or mock exosome (in a total volume of 200 μL of PBS) was injected into the tail vein three times a week for 5 weeks. Tumors were also measured three times per week. As mice injected with T47D mock cells did not develop tumors, mice were pre-treated with T47D-CXCR4-derived exosomes for three weeks before inoculation with T47D cells. After exosome pre-treatment, mice received the orthotopic inoculation of 1 × 10^6^ T47D mock cells. From this point, T47D-CXCR4-derived exosomes and T47D-mock-derived exosomes were injected for three weeks.

At the end of experiments, mice were euthanized and their tumors and organs were excised for subsequent examination. The presence of metastatic cells in lymph nodes was assessed by fluorescence detection using a TCS SP5 confocal microscope (Leica Microsystems) and analyzed by Leica Application Suite 2.02. Moreover, analysis of tumor growth and metastasis in mice with tumor generated by MDA-MB-231^FLuc^ was performed by *in vivo* and *ex vivo* BLI. For details, see Supplementary Material.

### Immunohistochemistry analysis

Immunohistochemistry and mitotic rate analysis of mice tissue sections were performed as described elsewhere [26].

### Patient samples, clinico-pathological parameters and follow-up

A series of 201 plasma samples from patients with breast cancer were provided by Biobank HUPH-M. The study was approved by the Research Ethics Board of the Hospital Universitario Puerta de Hierro-Majadahonda. The parameters obtained from the medical records of all the patients, prospective follow-up and therapies are mentioned in Supplementary Material. OS and DFS, defined as the period from time of diagnosis until death and the interval between diagnosis and first recurrence, respectively, were the study end-points. Exosome mRNA was detected in the plasma of 173 patients. The remaining cases were eliminated from the study.

### Statistical analysis

Comparisons between gene expression levels *in vitro* and functional effects on cancer cells were contrasted using the Student's *t*-test after evaluation of equality of variance with Levene's test. Results obtained from experiments requiring cultured cell lines and mice were expressed as mean ± standard deviation of at least three separate experiments. For survival study patients, we used optimal criterion value corresponding with the Youden's index to divide mRNA data of evaluated genes in high or low levels to discriminate between two populations (presence or absence of first relapse and death for SLE and OS, respectively). The relationship between the cumulative probability of OS and PFS, as well as analyzed predictors, was calculated by the Kaplan-Meier method, while significant differences between curves were evaluated with Mantel's log-rank test. To identify factors that might be of independent significance in influencing OS and DFS, the Cox proportional risk regression model was used. The clinical-pathological parameters were contrasted with the presence of evaluated mRNA by the χ^2^ test. In Box Plot representations, data were standardized by log_10_.

Two-tailed *p* values ≤0.05 were considered significant. *P*-values showed in figures are represented as: *, *p* ≤ 0.05; **, *p* ≤ 0.01; and ***, *p* ≤ 0.005. The mean and standard deviation (SD) were calculated by using Prism V (Graphpad Software), and error bars represent the SD. Statistical analysis used the SPSS, version 14.0.

## SUPPLEMENTARY MATERIALS AND METHODS, FIGURES AND TABLES




