# Supplementary material for: Exosomes enriched in stemness/metastatic-related mRNAS promote oncogenic potential in breast cancer
Source: Oncotarget. 2015 Oct 26;6(38):40575–87. doi: 10.18632/oncotarget.5818 (PMC4747353; doi:10.18632/oncotarget.5818)
Supplement: Supplementary file 1 [file oncotarget-06-40575-s001.pdf]

SUPPLEMENTARY MATERIALS AND METHODS

Cell culture

Cells were grown at 37°C in a humidified atmosphere of 5% CO_2. Human breast cancer cell lines (HCC38, MDA-MB-231 and T47D) were cultured in ATCC-formulated media (RPMI 1640; Lonza Group Ltd.), supplemented with 10% heat-inactivated Foetal Bovine Serum (FBS), 2 mM L-glutamine (Invitrogen), penicillin-streptomycin (10000 U/mL; Lonza Group Ltd.) and fungizone (0.25 µg/mL).

Exosome isolation from cell supernatants

Briefly, supernatant fractions collected were pelleted by centrifugation at 500 *g* for 10 minutes at 4°C to eliminate cells. The supernatant was centrifuged at 17,000 *g* for 20 minutes at 4°C, followed by passage through a 0.22-µm PVDF filter. Exosomes were then harvested by ultracentrifugation at 120,000 *g* for 90 minutes at 4°C (Optima™ MAX-XP, Beckman Coulter).

Exosome labeling

Labeled exosomes were washed in 9 mL of PBS, collected by ultracentrifugation and resuspended in RPMI-1640 supplemented with 10% exosome-depleted FBS. T47D cells were seeded in duplicate on 4-well chamber slides (Thermo Scientific) and incubated for 6 hours at 37°C with 100 µL of exosomes labeled with PKH67. The cells were subsequently fixed with 4% paraformaldehyde and washed twice in PBS. The samples were then incubated with mouse monoclonal antibody for E-Cadherin at a dilution of 1:20 (BD Biosciences) overnight at 4°C, washed in PBS and incubated with Alexa Fluor 546 anti-mouse in a 1:1000 dilution (Invitrogen Life Technologies) for 45 minutes at room temperature. Nuclei were stained with Topro-3 at a dilution of 1:1000 (Invitrogen Life Technologies) for 15 minutes and the sections were mounted with PBS glycerol.

Images were collected with a TCS SP5 confocal microscope (Leica Microsystems) equipped with 40× HCX PL APO (1.25–1.52 numerical aperture) oil-immersion optics. Images were captured with a scanning speed of 400 Hz and image resolution of 1,024 × 1,024 pixels and then analyzed by Leica Application Suite 2.02.

Protein extraction and western blot analysis

Lysates were cleared by centrifugation and the supernatant was used for Western blot. Protein extracts were denatured in 2× SDS buffer at 95°C, separated in 10% SDS-polyacrylamide gel electrophoresis and transferred by the iBlot System (Invitrogen). Membranes were blocked and incubated with mouse monoclonal antibodies for CD63 or CD81 at a dilution of 1:50, and rabbit polyclonal antibody for Calnexin as loading control (1:50 dilution). Anti-mouse IRDye 800 CW infrared polyclonal secondary antibody was used at a dilution of 1:2000 (Rockland). The band intensities were quantified by densitometry, using Odyssey Infrared Imaging System (LI-COR Biosciences). The Bio-Rad Protein Assay was used for protein quantification.

Flow cytometry

HCC38, MDA-MB-231, CXCR4-T47Dand T47D mock cells were incubated with monoclonal antibodies rat anti-human CD44 and mouse anti-human Integrin alpha 6 (CD49f) conjugated with fluorescein isothiocyanate (FITC) (Abcam). Mouse IgG1-FITC irrelevant antibody (Abcam) was used as negative control of staining.

Animals and tumor xenografts

Mice were kept under anesthesia during all manipulations and all efforts were made to minimize suffering. Anesthesia was induced with 2.5% isofluorane in 100% oxygen at a flow rate of 1 L/min, and maintained with a 1.5% mixture at 0.5 L/min. Animals were housed under pathogen-free conditions and were given irradiated food and autoclaved water *ad libitum*.

In vivo and *ex vivo* bioluminescence imaging

The mice were anesthetized using inhaled isoflurane, as described above, injected i.p. with 125 mg/kg (100 µL) D-luciferin dissolved in PBS or with 900 mg/kg (100 µL) coelenterazine-based compound dissolved in PBS, and placed on a thermostatic bed. Bioluminescence imaging was collected with 1 minute integration time in F^{Luc} imaging, and pseudocolor representations of light intensity were superimposed over the grayscale reference image acquired at low light (20 ms). An average of 6 kinetic BLI acquisitions was collected after substrate injection to confirm a peak of photon emission. For quantitation of the light detected, regions of interest were drawn and the light emitted from each region was recorded by measuring the total number of photons per second (total flux) after background subtraction. At the end of each experiment anesthetized mice were euthanized and lymph nodes and various organs (lung, brain and spleen) were rapidly harvested and processed. Organs were placed in PBS containing coelenterazine-based compound and individually and immediately scanned for the presence of F^{Luc} bioluminescent metastatic cells for 1 minute of integration time, as described above.

Mice were imaged using the high-resolution charge-coupled-device (CCD) cooled digital camera ORCA-2BT (Hamamatsu Photonics France), and Hokawo software (Hamamatsu Photonics).

Patient samples, clinico-pathological parameters and follow-up

The following parameters were obtained from the medical records of all the patients: age; tumor size; lymph nodes metastases (LNM); presence of steroid receptors; menstrual status; pathological stage (tumor-node-metastasis classification); histological grade (Bloom-Richardson system); proliferative index (Ki67); c-erbB2, p53 and bcl2 status; histological type; vascular/lymphatic invasion; systemic treatment administered; and dates of recurrence or death (when applicable), and follow-up. The steroid receptor content; Ki67, p53, c-erbB2 and bcl2 expression were determined by immunohistochemical procedures.

Prospective follow-up, starting after surgery and diagnosis, was based on a regular (every 3 months during the first year, every 6 months during the second year, and then yearly until relapse) clinical, biochemical and radiological examination (chest X-ray, mammography and other areas as clinically indicated), including bone scan and liver ultrasound when liver function was impaired. Hormonal therapy was administered to 74.8% of patients (tamoxifen 20 mg or Anastrozol 1 mg, daily) and chemotherapy to 47.6% of patients (60 mg/m^2 Adriamycin i.v. on day 1 and 600 mg/m^2 cyclophosphamide i.v. on day 1).

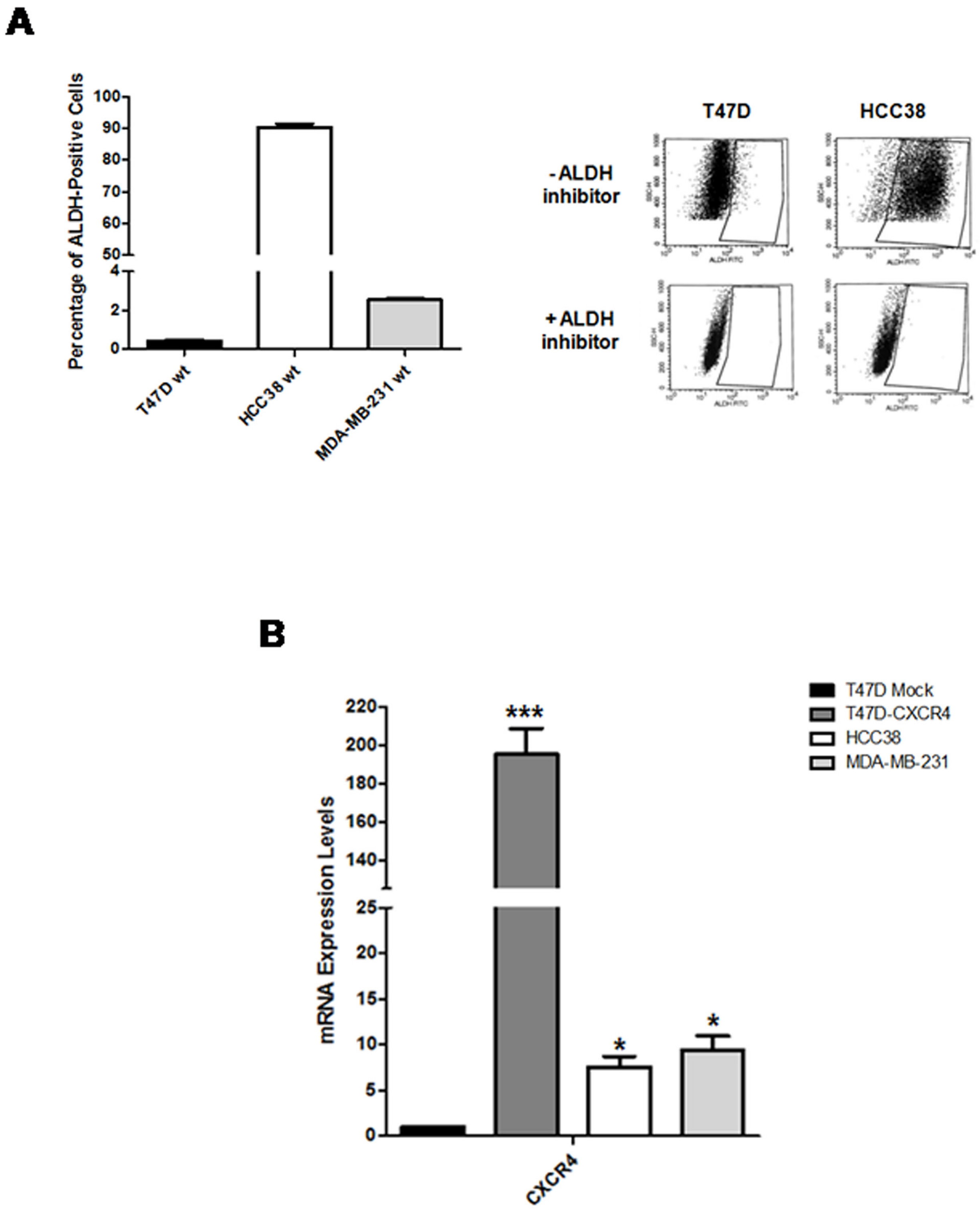

Supplementary Figure S1: A. ALDH activity in HCC38, MDA-MB-231 and T47D cell lines. Aldefluor™ fluorescence versus SSC dot plot of human breast cell lines. An inhibitor of ALDH was used as negative control for background fluorescence for each sample. **B.** CXCR4 expression in CXCR4-cells and human breast cancer cell lines (*$p \leq 0.05$; **$p \leq 0.01$; and ***$p \leq 0.005$). As expected, CXCR4 was significantly increased in CXCR4-T47D cells compared with T47D mock cells.

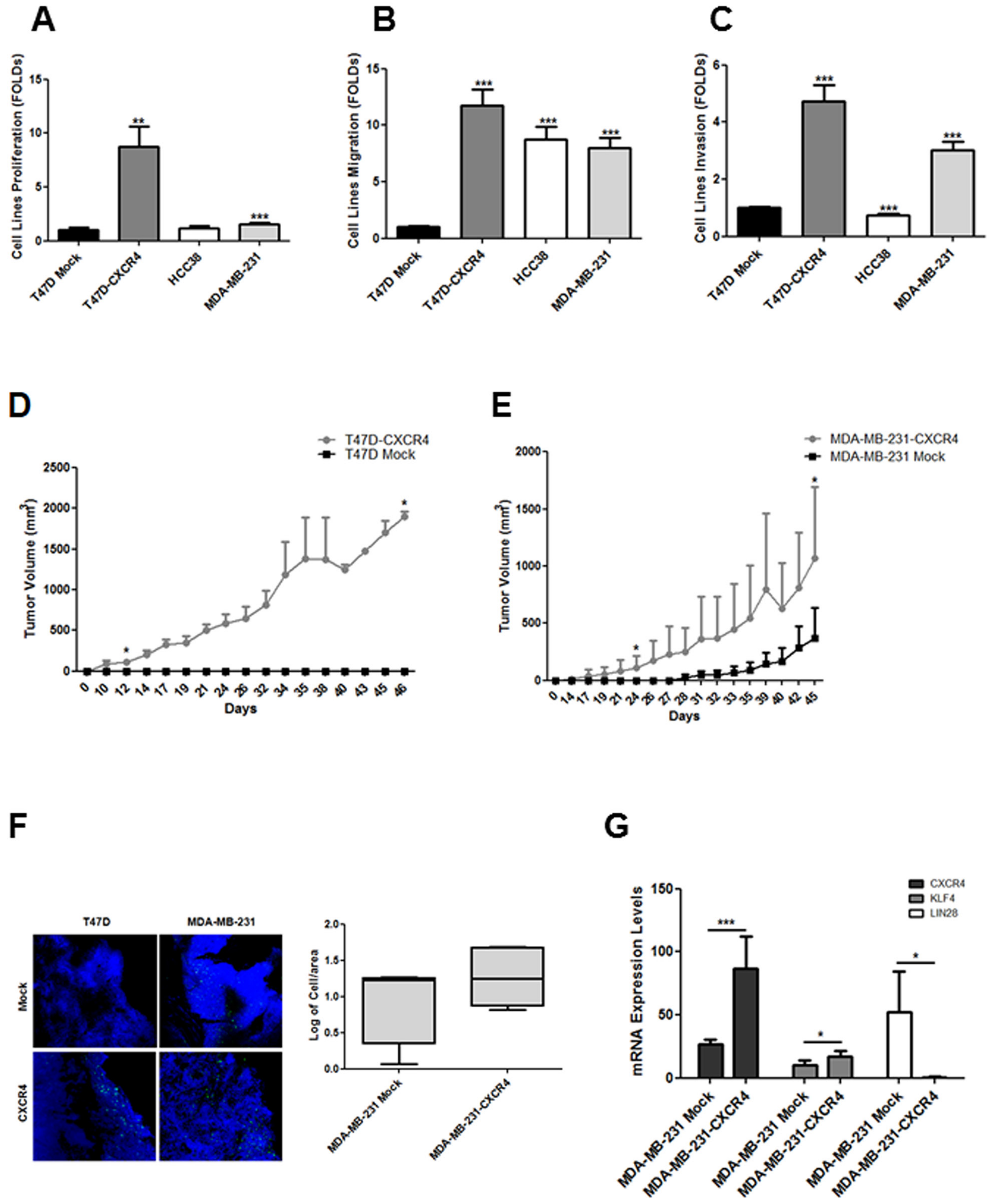

Supplementary Figure S2: MDA-MB-231 and CXCR4-T47D cells showed higher **A.** proliferation, **B.** migration and **C.** invasion rates than T47D mock cells at 48, 24 and 72 hours, respectively (**$p \leq 0.01$; and ***$p \leq 0.005$). Evolution of tumor growth in immunodeficient mice orthotopically injected with **D.** CXCR4-T47D and **E.** CXCR4-MDA-MB-231 cells and their respective mock cells (*$p \leq 0.05$; $N = 5$). As expected, CXCR4 is involved in tumor growth. **F.** Confocal microscopy images (10X magnification) of excised lymph nodes (Topro-3, blue) from mice showing metastasis potential of CXCR4-cells (GFP^{+}, green). Quantification of the total number of MDA-MB-231 cells is shown on the right. Mice with tumors derived by CXCR4-cells showed increased capacity of metastasis in lymph nodes. **G.** mRNA expression of stemness-related markers in primary tumors generated in immunodeficient mice orthotopically injected with CXCR4-MDA-MB-231 cells (*$p \leq 0.05$; and ***$p \leq 0.005$). Several markers were increased in primary tumors generated by CXCR4-cells.

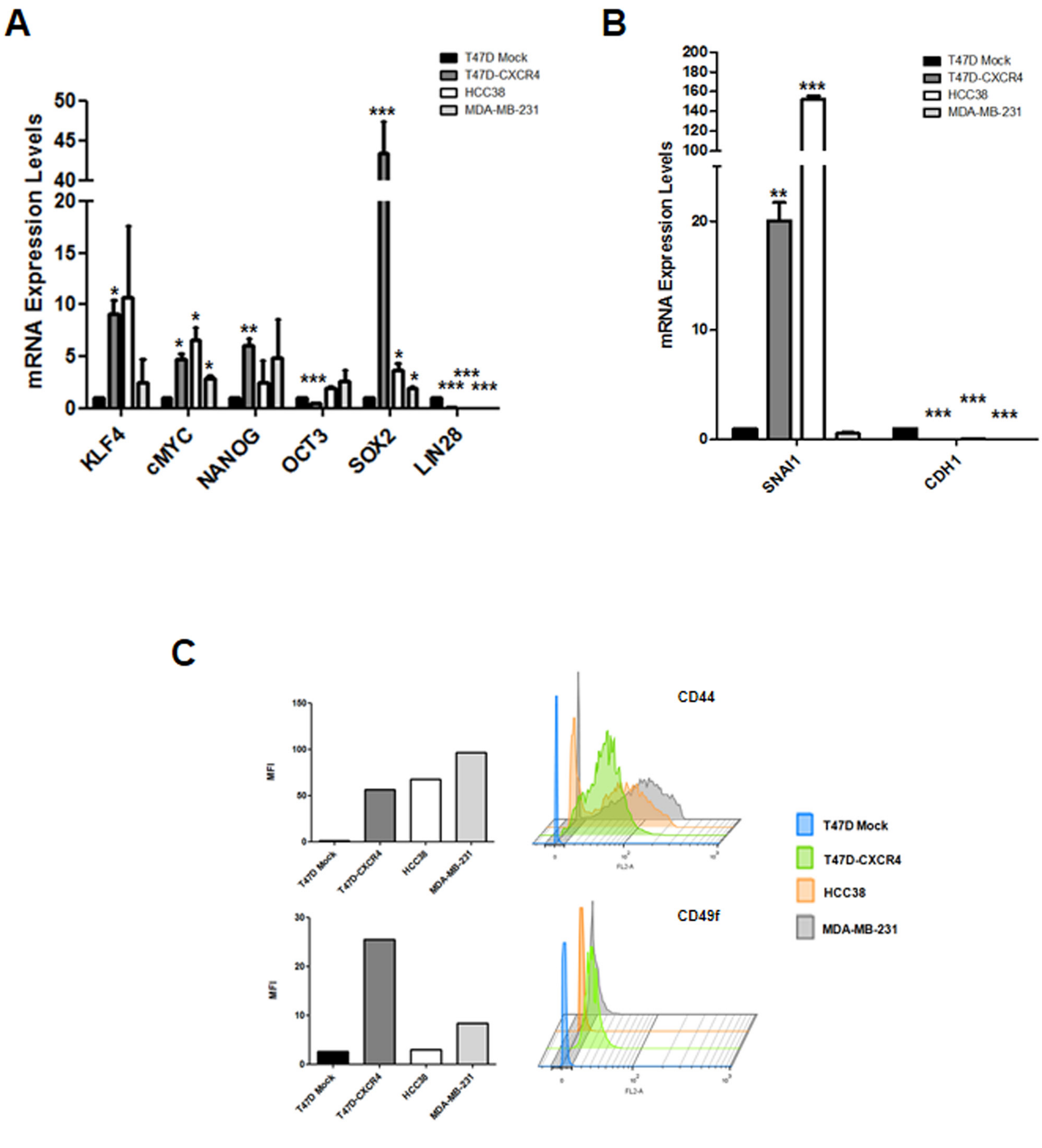

Supplementary Figure S3: mRNA expression of **A.** stemness and **B.** EMT-related markers in CXCR4-transfected cells and tumor cell lines (*$p \leq 0.05$; **$p \leq 0.01$; and ***$p \leq 0.005$). Data show that these markers were associated with stemness-, metastatic- and CXCR4-cells. **C.** Analysis of subpopulations of breast cancer stem cells through of antigens phenotypes ($CD44^{high}$ and $CD49f^{high}$) by FACS analysis in CXCR4-cells and human breast cancer cell lines. CXCR4-cells, HCC38 and MDA-MB-231 showed increased expression of these surface antigens.

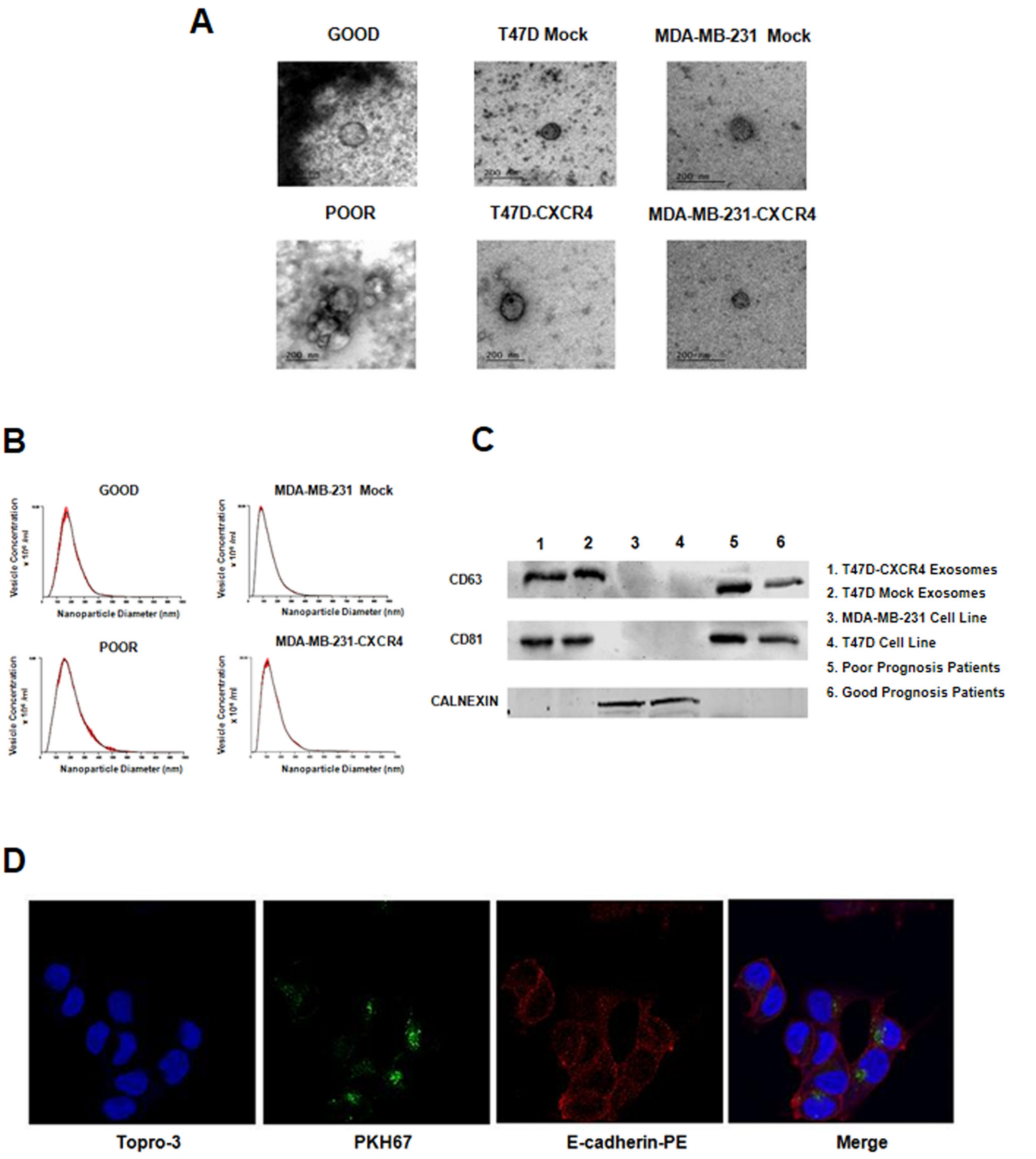

Supplementary Figure S4: Characterization of isolated exosomes from good and poor prognosis patients and from tumor cells by: **A.** transmission electron microscopy; **B.** nanoparticle tracking analysis; and **C.** immunoblotting of exosomal-related proteins CD63, CD81 and Calnexin. **D.** Uptake of PKH67-labeled exosomes by T47D cells. In the confocal microscopy image (10X magnification), Topro-3 (blue) was used to detect nuclei of cells, E-Cadherin (red) to label cell membrane, and PKH67 (green) to label the exosomes.

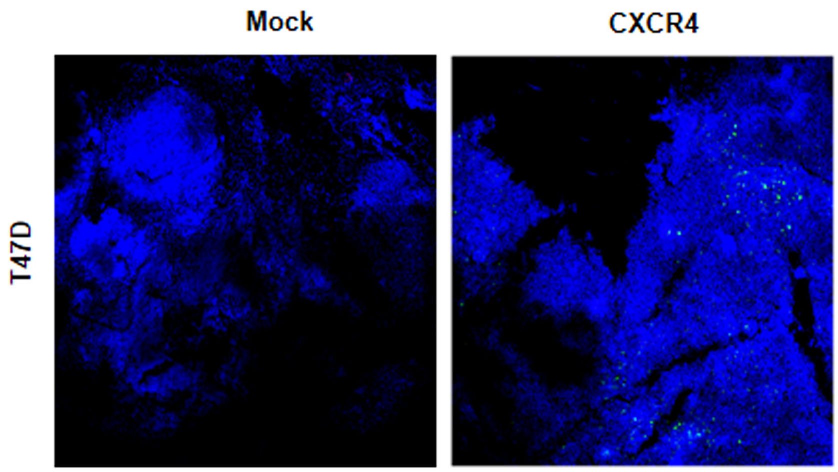

Supplementary Figure S5: Metastasis after fat pad injection of T47D cells in mice treated with CXCR4-T47D- or mock-T47D-derived exosomes. Confocal microscopy images (10X magnification) of lymph nodes (Topro-3, blue) removed from mice showing metastasis potential of CXCR4-cells (GFP$^+$, green).

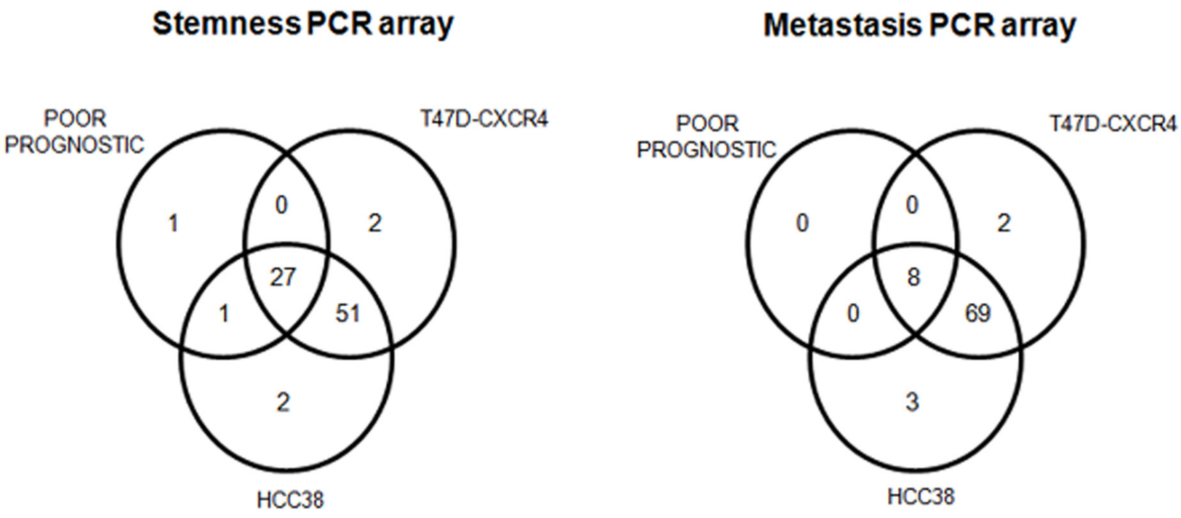

Supplementary Figure S6: Number of mRNAs with high levels in exosomes of CXCR4-T47D and HCC38 cell lines, and of pool of plasma from poor prognostic patients analyzed by quantitative PCR arrays.

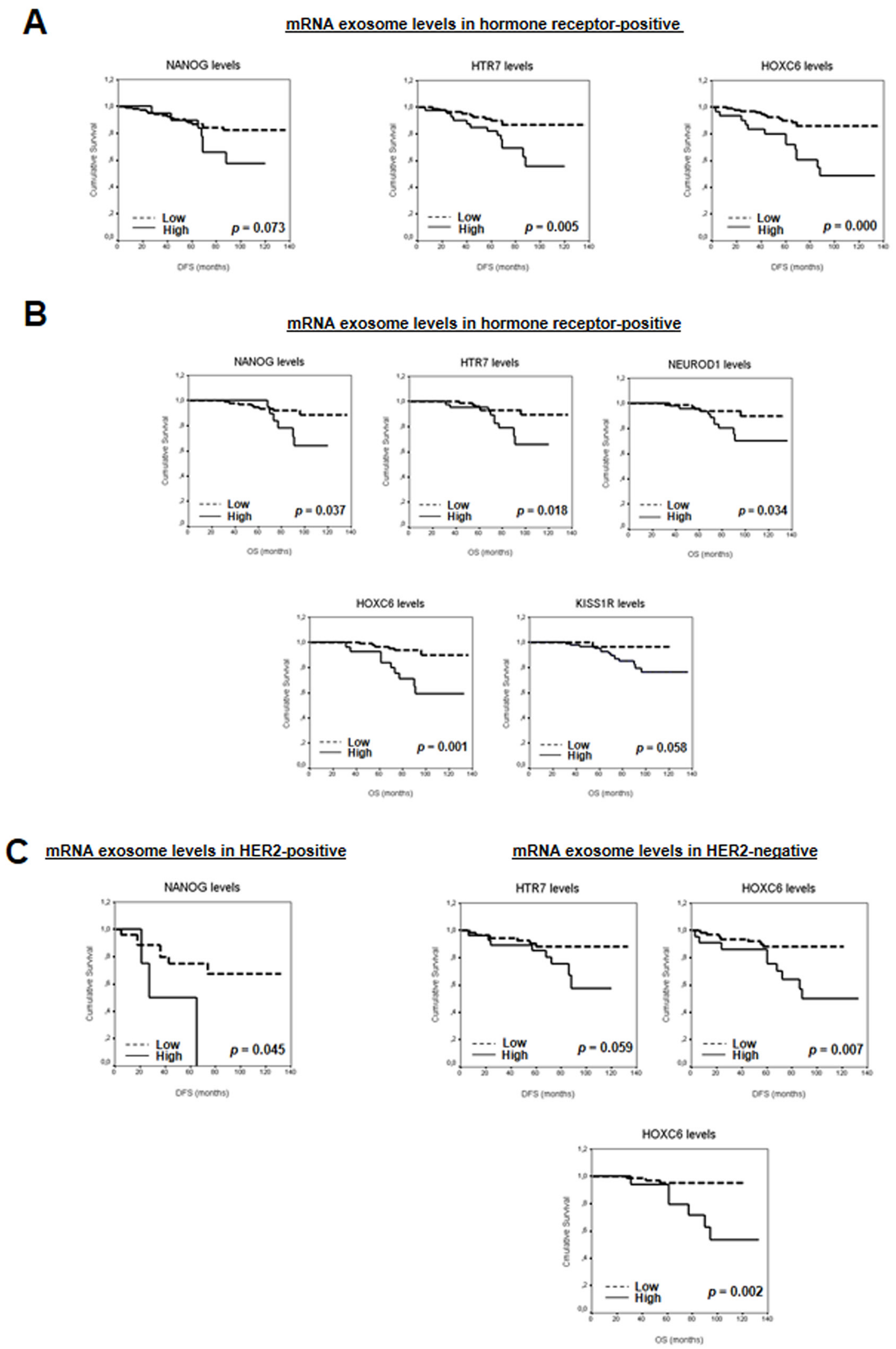

Supplementary Figure S7: A. Kaplan-Meier DFS and **B.** Kaplan-Meier OS curves in relation to levels of validated mRNA in exosomes from patients classified on the basis of hormone receptors. **C.** Kaplan-Meier DFS and OS curves in relation to levels of validated mRNA in exosomes from patients classified on the basis HER2 status.

Supplementary Table S1: Top ten stemness and metastatic-related mRNAs with highest levels in CXCR4-T47D and HCC38 cell lines, and in pool of poor prognostic patients.

Supplementary Table S2: Analysis of DFS and OS with the validated mRNA levels in exosomes from (A) total series patients, and patients classified on the basis of (B) hormone receptors and (C) HER2 status.

mRNA		% DFS (95% CI) 100 months	*P*	% OS (95% CI) 100 months	*P*
TOTAL SERIES (A)	***HTR7***<br>Low<br>High	 84.07% (76.56–91.57%)<br>53.81% (36.66–70.96%)	0.003	 86.39% (78.04–94.73%)<br>65.31% (47.37–83.24%)	0.032
	NEUROD1<br>Low<br>High	 81.43% (72.37–90.48%)<br>63.92% (49.96–77.87%)	0.027	 88.25% (79.76–96.73%)<br>65.86% (82.04–49.67%)	0.032
	HOXC6<br>Low<br>High	 80.1% (72.4–87.7%)<br>53% (33.2–72.7%)	0.019	 82.3% (72.1–92.5%)<br>56.5% (35.1–77.8%)	0.013
	NANOG<br>Low<br>High	 78.6% (70.88–86.36%)<br>54.05% (31.25–76.84%)	0.076	 86.1% (78.4–93.7%)<br>60.3% (39.2–81.4%)	0.049
	KISS1R<br>Low<br>High	 81.27% (67.86–94.68%)<br>71.23% (61.67–80.79%)	NS	 93.47% (86.27–100%)<br>73.49% (62.31–84.66%)	0.064
HORMONE RECEPTOR-POSITIVE (B)	***HTR7***<br>Low<br>High	 86.72% (78.95–94.48%)<br>55.95% (35.17–76.72%)	0.005	 89.47% (80.55–98.38%)<br>65.78% (45.39–86.16%)	0.018
	NEUROD1<br>Low<br>High	 83.72% (74.25–93.18%)<br>68.26% (52.24–84.27%)	NS	 89.82% (80.39–99.24%)<br>70.44% (53.50–87.37%)	0.034
	HOXC6<br>Low<br>High	 85.8% (78.1–93.4%)<br>48.7% (27.7–69.6%)	<0.001	 90.1% (81.4–98.7%)<br>59.2% (37.8–80.5%)	0.001
	NANOG<br>Low<br>High	 82.29% (74.11–90.46%)<br>57.57% (32.85–82.28%)	0.073	 88.61% (80.18–97.03%)<br>40% (0–98.13%)	0.037
	KISS1R<br>Low<br>High	 81.59% (66.49–96.68%)<br>76.26% (66.14–86.37%)	NS	 96.67% (90.24–100%)<br>76.40% (64.42–88.37%)	0.057

(*Continued*)

mRNA		% DFS (95% CI) 100 months	*P*	% OS (95% CI) 100 months	*P*
HER2 (C) POSIT NEGAT	***HTR7*** Low High	88.04% (78.98–97.09%)57.49% (31.89–87.08%)	0.059	89.68% (81.03–98.32%)69.69% (41.74–97.63%)	NS
	HOXC6 Low High	88.1% (79.6–96.5%) 50% (25.9–74.1%)	0.007	95.3% (90.2–100%) 53.8% (25.7–81.8%)	0.002
	NANOG Low High	67.44% (46.38–88.49%) 0% (0%)	0.045	81.85% (65.32–98.37%) 33.33% (0–86.68%)	NS

Supplementary Table S3: Analysis of DFS and OS with "stemness and metastatic signature" levels in exosomes from total series patients, and in exosomes from patients classified on the basis of hormone receptors and HER2 and HER2 status.

	mRNA	% DFS (95% CI) 100 months	*P*	% OS (95% CI) 100 months	*P*
**TOTAL SERIES**	**signature** Low High	82.2% (74.2–90.1%) 54.1% (36.2–72.1%)	0.002	90.3% (83.2–97.5%) 54.8% (35%-74.6%)	<0.001
**HR-positive**	**signature** Low High	85.4% (77.3–93.6%) 55.7% (33.3–78.0%)	0.004	92.79% (85.16–100%) 56.98% (35.06–78.89%)	<0.001
**HER2-negative**	**signature** Low High	85.25% (74.27–96.22%) 56.62% (29.4–83.84%)	0.035	94.62% (86.68–100%) 58.81% (29.80–87.81%)	0.024

Supplementary Table S4: Associations between clinicopathological characteristics and "stemness and metastatic signature" levels in exosomes of breast cancer patients

	Stemness and metastatic signature		*P*
	Low levels (%)	High levels (%)	
**Age**			
(< 50 years	27.7	6.9	0.008
(≥ 50 years	39.3	26	
**VLI**			
No	45.5	19.5	NS
Yes	21.4	13.6	
**Tumor size**			
≤ 2 cm	36.1	16.6	NS
> 2 cm	31.4	16	
**LNM**			
No	39.3	18.4	NS
Yes	27	33.7	
**Grade**			
I	14.8	7.1	NS
II	25.8	12.3	
III	25.2	14.8	
**ER**			
Negative	14.5	7.2	NS
positive	52.4	25.9	
**PR**			
negative	19.5	9.1	NS
positive	48.2	23.2	
**HER2**			
negative	49.6	24	NS
positive	19.8	6.6	
**p53**			
negative	50	20.3	NS
positive	17.5	12.6	
**Ki67**			
negative	22	8.4	NS
positive	45.8	23.9	
**Bcl-2**			
negative	16.7	8	NS
positive	52	23.3	
**Stage**			
I, II, IIIA	65.1	29	0.056
IIIB, IV	5.8	3.6	
**RT**			
responsive	66.9	31.2	NS
non-responsive	1.9		

(*Continued*)

	Stemness and metastatic signature		*P*
	Low levels (%)	High levels (%)	
**Relapse**			
No	57.8	20.2	<0.001
Yes	9.2	12.7	
**LRS**			
Without disease	56.7	19.9	
With disease	2.9	4.1	<0.001
Death	7	12.3	

VLI, vascular and lymphatic invasion; LNM, lymph node metastases; ER, oestrogen receptor; PR, progesterone receptor; RT, response to treatment; LRS, last revision status; NS, no significant.

Supplementary Table S5: Sequence of primer pairs and conditions for mRNA of EMT-related genes, *CXCR4*, stemness-related genes and control gene (*SDHA*, succinate dehydrogenase complex subunit A)

mRNA	Primers Sequence	AT (°C)
SDHA	5′TGGGAACAAGAGGGCATCTG’F<br>5′CCACCACTGCTCAAATTCATG’R	59
SNAI1	5′CACTATGCCGCGCTCTTTC’F<br>5′ GGTCGTAGGGCTGCTGGAA’R	68
CDH1	5′CTACACGTTCACGGTGCCC’F<br>5′GTCCTTTGTCGACCGGTGC’R	60
CXCR4	5′TATGACTCCATGAAGGAACCCTGT’F<br>5′AGCCTGTAACTTGTCCGTCATGC’R	63
KLF4	5′CAAGTCCCGCCGCTCCATTACCAA’F<br>5′CCACAGCCGTCCCAGTCACAGTGG’R	59
cMYC	5′TGGATTTTTTTCGGGTAGTGG’F<br>5′TCCAGATATCTTCGCTGGG’R	59
NANOG	5′CTAAGAGGTGGCAGAAAAACA’F<br>5′CTGGTGGTAGGAAGAGTAAAGG’R	59
OCT3	5′ACTGCAGCAGATCAGCCACATCG’F<br>5′ATCCTCTCGTTGTGCATAGTCGC’R	63
SOX2	5′ACACCAATCCCATCCACACT’F<br>5′GCAAACTTCCTGCAAAGCTC’R	59
LIN28	5′CGGGCATCTGTAAGTGGTTC’F<br>5′CAGACCCTTGGCTGACTTCT’R	59

F, forward; R, reverse.
